# Differential Diffusion of Helium Isotopes in Glass, Quantum-tunneling ^3^He Enrichment, and Portable ^3^He/^4^He Monitoring of Mantle Processes

**DOI:** 10.1038/s41598-019-41360-5

**Published:** 2019-03-26

**Authors:** Gary M. McMurtry, James R. DeLuze, David R. Hilton, James E. Blessing

**Affiliations:** 1 Department of Oceanography, School of Ocean and Earth Science and Technology, University of Hawaii, Manoa, Honolulu, HI 96822 USA; 2 Fusion Energy Solutions of Hawaii, 611 University Avenue, Apt. 301, Honolulu, HI 96826 USA; 3 Scripps Institution of Oceanography, University of California, San Diego, 9500 Gilman Drive, La Jolla, CA 92093-0244 USA; 4 MKS Instruments, Inc., Mass Spectrometry Solutions, 3635 Peterson Way, Santa Clara, CA 95054 USA

## Abstract

While studying the scientific and engineering aspects of a field-portable ^3^He/^4^He ratio detector, we found elevated ratios at comparatively lower temperatures that appear to result from differential diffusion of these isotopes in pure quartz glass. The ^3^He enrichment relative to ^4^He in lab air, expressed as the ratio R and normalized to the accepted ^3^He/^4^He ratio of 1.40 E-06 (R_a_), ranges from peak values of about 200 to 600 in dry static samples. Even at the maximum classical ^3^He/^4^He diffusivity ratio of 1.15, the expected R would be only 1.61 E-06. Within a narrow temperature window, the air value in our experimental set up with pure quartz glass can range from about 2.70 to 8.20 E-04, or nearly 1000 times the expected enrichment based upon classical fractionation. When plotted versus temperature, the narrow ^3^He net partial pressure peak reveals at least three sharper embedded peaks that may be quantized vibrational entrance states in quartz glass which are temperature specific. This discovery has implications for relatively low-energy industrial enrichment of scarce ^3^He from natural sources on Earth, and for radiogenic and cosmogenic helium dating assumptions in natural glasses. It also has bearing upon designs for field portable ^3^He/^4^He ratio detectors aimed at earthquake and volcanic eruption studies, and monitoring of nuclear sites.

## Introduction

Helium is known to diffuse through glass as a function of temperature, pressure, and purity of the silica glass matrix^1–3^. The fractionation of the ^3^He isotope of helium in glass relative to its heavier and far more abundant isotope, ^4^He, is less well understood. Trull^4^ studied the fractionation of these isotopes during diffusion in natural glasses and silicate minerals and found that the enrichment of ^3^He, expressed as the diffusivity isotope ratio D ^3^He/D ^4^He was only 1.08 and not compositionally dependent, whereas classical mechanics predicts as much as 1.15 based upon their mass ratio. Earlier work by Shelby^5^ found that the diffusivity isotope ratio for this pair in vitreous silica was as high as 1.12 at temperatures approaching 600 °C, and ascribed the effect to a quantum correction first proposed by LeClaire^6^. Here we report experimental data that indicates the temperature and time-dependent diffusivity ratio, D ^3^He/D ^4^He can be far greater than 1.15 in pure quartz glass at temperatures below 300 °C. Quantum tunneling (e.g. ref.^7^) over short distances aided by interstice channels is the most plausible mechanism to explain these large ^3^He enrichments at comparatively low temperature.

## Results

**Figure 1:**
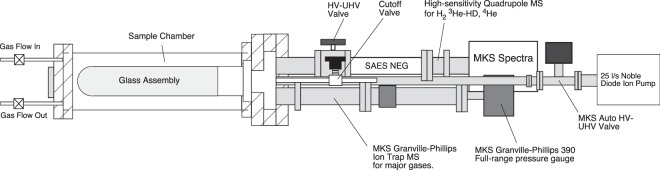
Schematic diagram of an experimental set-up with the “Albert” bench-top prototype of a field-portable ^3^He/^4^He instrument. Major components are labeled.

**Figure 2:**
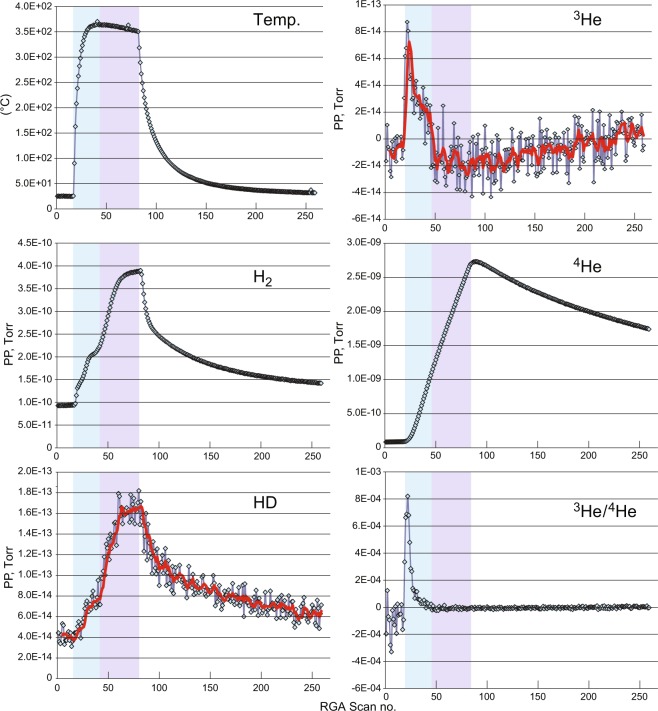
Plots of temperature, measured H_2_ partial pressure (PP), AIMS-calculated HD PP, AIMS-calculated ^3^He PP, measured ^4^He PP, and ^3^He/^4^He ratio trends for a static laboratory air heat ramp to maximum 370 °C from ambient lab temperature. The data were generated by the instrument described in Fig. 1 in communication with a laptop PC that commonly used an MKS RGA program recipe and the standard MKS VQM software. Temperatures fed into the RGA and the RGA scans were averaged over 57 seconds. The HD and ^3^He curves are 5-point moving averages. (HD in these plots actually represents the combined abundance of HD, H_3_ and any environmental ^3^H that may be present. See Supplementary Information.) Blue shading represents temperature region where ^3^He, H_2_ and HD rapidly rise; purple shading represents temperature region where H_2_ and HD continue to rise. In contrast, ^4^He slowly rises linearly through both these temperature regions and peaks shortly after power to the heater has stopped. Negative PP values for ^3^He are likely caused by signal suppression near its limit of detection from the ^4^He build.

Experiments were conducted on a compact, elongate vacuum apparatus described in the Methods (Fig. 1). The results presented herein were recorded throughout a typical heating cycle, e.g., from ambient lab temperature to a maximum temperature usually reached before one hour of applied power and then cooled to near ambient temperature. The trends of a typical, repeatable static lab air heat ramp are shown in Fig. 2. Two portions of this ramp are highlighted: one (blue) showing enrichments of ^3^He, HD and H_2_ and a second portion (purple) showing continued gains in HD and H_2_ partial pressure (PP) as the maximum temperature peaks and begins to slowly decrease at the same or slightly higher power level applied to the heating element, i.e., as the glass cools. Heating power was turned off at the end of these portions. In contrast to the ^3^He, HD and H_2_ trends, ^4^He rises linearly and peaks shortly after heater power ceases. Expressed as the ^3^He/^4^He ratio, upon heating a maximum of 8.20 E-04 is seen at a temperature of 263 °C, the peak ranging from ca. 130° to 370 °C (Fig. 2). Such high ratios exceed those of interplanetary dust particles or the Moon’s regolith^8,9^. As we demonstrate below, this enrichment results from differential diffusion of ^3^He and ^4^He in heated quartz glass. Some diffusion of these gases may also occur in the lower-temperature glasses used to fuse the quartz glass to metal, such as Pyrex™ and Kovar™, but these glasses contain metals that block the open Si-O ring structure in pure silica glass, leading to much lower diffusion rates (c.f. ref.^3^).

**Figure 3:**
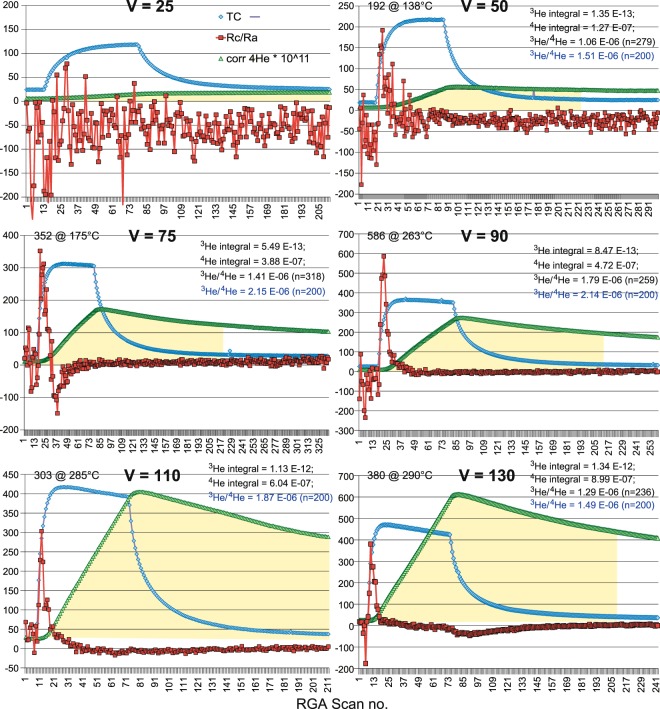
Static air temporal trend plots of thermocouple temperature (TC, °C), corrected ^4^He partial pressure (PP, units of Torr), and R_c_/R_a_, where the corrected ^3^He/^4^He ratio of the sample (R_c_) has been normalized to the accepted air ratio of 1.40 E-06 (R_a_). These corrections account for variable peak response of the mass spectrometer to changes made to the filament ionization level and the electron multiplier detector setting during the analysis. Sample numbers represent ca. 1-minute averages of the scanning MS response and the TC temperature. Heater power levels are indicated as voltage settings of the Variac™ autotransformer used. V = 25, 50, 75, 90, 110 and 130 panels correspond to applied AC power levels of 28, 100, 220, 320, 470 and 625 W, respectively. Yellow areas represent net ^4^He PP accumulated for 200 minutes; corresponding ^3^He/^4^He ratios are denoted in blue ink. In all cases, the ^3^He/^4^He values reported were calculated as ratios of net PP accumulations. Numbers at peak temperatures refer to these calculated ratios.

Figure 3 presents temporal trend plots of temperature, ^4^He partial pressure, and R_c_/R_a_ (rationale for this ratio terminology given in caption) for the case of static flow at six progressively higher power settings. In all cases, heating power was applied for one hour and data recorded until the sample chamber cooled to about 10 °C above ambient, which ranged from 23° to 25 °C. Net isotope integrals represent the integrated peak response above their respective background levels. Yellow shaded areas represent the integrated, corrected ^4^He partial pressure for 200 minutes (arbitrarily set by the V = 110 run). The ^3^He/^4^He ratio in blue is the corresponding value for 200-minute integrations. Between applied AC power levels of 28 to 100 W, ^3^He net partial pressure builds in the vacuum by diffusion through the glass within the first ca. 20–30 minutes of the heat ramp, as shown by the R_c_/R_a_ trend. High R_c_/R_a_ values are indicated at ^3^He peak diffusion temperatures. ^4^He net partial pressure takes considerably longer to build in the instrument vacuum, increasing with power level and maximum temperature (Fig. 3). The air ratio is achieved in about 200 minutes at V = 130, but takes over 300 minutes at V = 75. Other power settings show intermediate and longer-period results; at V = 25, very little ^3^He relative to ^4^He is diffusing through the glass. At higher power settings, above V = 50, the ^4^He conductance increases with increased maximum glass temperatures as the run progresses. The result is that by the end of the run, the ^4^He partial pressure overcomes the low-temperature ^3^He conductance advantage due to the much greater abundance of ^4^He. The increased abundance of ^4^He then raises the noise floor of the quadrupole to above the ^3^He signal (see panel V = 130, Fig. 3).

Except for the two lowest power ramps, heat ramps were run progressively, with ≥1-hour exposure to the ion pump to reduce the prior sample’s vacuum pressure, usually by greater than an order of magnitude. Subsequent heat ramps with the sample chamber under vacuum and with dry tank N_2_ as a pressure compensation have shown a reservoir effect for helium isotopes in glass, which can be cleared after 1–2 hours of heating to >400 °C (e.g. ref.^10^). This effect may have added ^3^He and ^4^He to the progressive heat ramps in Fig. 3, but does not change the overall results presented.

## Discussion and Conclusions

The ^3^He enrichment relative to ^4^He in lab air, expressed as the ratio R and normalized to the accepted ^3^He/^4^He ratio of 1.40 E-06 (R_a_), ranges from peak values of about 200 to 600 in dry static samples (Figs. 2 and 3). Even at the maximum classical ^3^He/^4^He diffusivity ratio of 1.15, the expected R would be only 1.61 E-06. Within a narrow temperature window, the air value in our experimental set up with pure quartz glass can range from about 2.70 to 8.20 E-04, or nearly 1000 times the expected enrichment based upon classical fractionation. These enormous enrichment values, and their unexpected variance with temperature are, however, consistent with a quantum tunneling (QT)-enhanced fractionation of ^3^He/^4^He in similar molecular ring-structure matrices (e.g., refs.^11–15^). However, as indicated in Fig. 3, integration over the duration of the experimental heat ramp produces ^3^He/^4^He ratios that closely approach the accepted value for air. Additional supporting data to the trends presented in Figs. 2 and 3 are presented in the supplementary information section.

The tunneling diffusion that creates the very high ^3^He/^4^He fractionation observed in modified graphene is essentially a surface phenomenon involving a few atomic layers and cooled to very low temperatures, but occurs up to room temperature^11–15^. It is similar to the resonant tunneling of electrons over distances of about 100 atomic layers in doped semiconductors that allow fast switching in tunneling diodes (e.g. ref.^16^). The exact mechanism of resonant tunneling-assisted diffusion in quartz glass up to 2 mm in thickness, as in this study, is presently unknown, but the vitreous silica contains migration paths for atoms to pass from interstice to interstice through channels formed by Si-O rings^10^. Indirect evidence for such structural interstices housing helium is the need for glass heating to high temperature under pressurized nitrogen gas to effectively clear the glass volume of prior helium loads in our experiments. The diffusion mechanism may be similar to that proposed for hydrogen atom diffusion through amorphous and polycrystalline ice, where tunneling diffusion may occur for a short distance within each single crystal or domain, whereas it should be highly suppressed for long-distance diffusion beyond steps and grain boundaries^17^.

**Figure 4:**
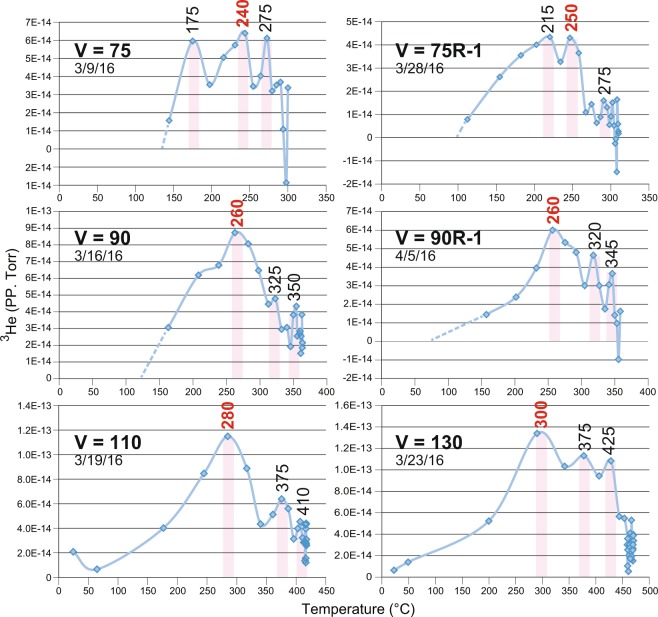
Plots of corrected ^3^He PP versus temperature for heat ramp power conditions V = 75, V = 75R-1 (replicate), V = 90, V = 90R-P (replicate, pumped air flow), V = 110 and V = 130. Dates in each panel refer to the run dates at the constant voltage indicated. At least three ^3^He peaks are identified within each heat ramp, corresponding to average temperatures of 248 ± 46 °C, 314 ± 59 °C, and 348 ± 65 °C. Red numbers denote the most intense and consistent peaks. Unfortunately, in these early experiments, the poor sampling rates of both ramp temperature and mass spectrometer scan averages caused peak resolution uncertainty. What is most relevant to our conclusions is the ^3^He peaks occur at progressively higher temperatures at higher power settings, due to shorter dwell times at a given temperature. The resolution can potentially be increased with longer heat ramps and increased sampling rates.

When plotted versus temperature, the narrow ^3^He net partial pressure peak reveals at least three sharper embedded peaks (Fig. 4), corresponding to mean temperatures of 248 °C, 314 °C, and 348 °C within the six heat ramps shown in Fig. 3. These three sharper peaks are temperature specific and likely responsible for most of the differential diffusion effects observed. Because glass has high thermal insulation properties, the actual temperatures at which selective ^3^He transmission phenomena occur are believed to be lower than recorded data. The increased temperatures observed with higher constant power settings (higher voltages) which result in steeper slopes of temperature rise demonstrate aggravation of this thermal lag effect. These peaks are interpreted as multiple resonant tunneling temperature states in quartz glass for quantum diffusion of ^3^He (c.f., refs.^16,18^). Higher sampling rates should improve the resolution of these temperature peaks, which we anticipate to be very narrow, and in the range of 140° to 300 °C. ^3^He shares chemical assignation to ^4^He but during diffusion through glass it resembles the better-studied quantum tunneling of hydrogen through solids (e.g. ref.^17^) rather than the much slower diffusion of ^4^He.

Previous investigations established that the diffusion of helium and its isotopes increases with increasing temperature in both natural and synthetic silicate glasses^4,19,20^. These investigations also suggested that there is an increasing fractionation of ^3^He to ^4^He with temperature, which is opposite in both sign and magnitude with our experimental findings in pure quartz glass. Craig and Lupton^21^ and Rison and Craig^22^ noted the loss of magmatic helium and large changes in the ^3^He/^4^He ratios of natural glasses relative to vesicles in young submarine basalts (with some alkalic basalts containing thin glass vesicle walls of a mm or less thickness) that they ascribed to low-temperature (ca. 2°–4 °C) exchange with seawater. These changes may have at least been aided by the extreme fractionation we have found due to QT effects in pure quartz glass. When using the ^3^He/^4^He ratios of natural glasses as a dating tool^21,23^, the assumption of closed system behavior may have yet another complication to address.

The QT effects we have found in pure quartz glass were an inadvertent discovery made while studying the scientific and engineering aspects of a field-portable ^3^He/^4^He ratio detector, on a pathway toward continuous monitoring of these ratios in both natural gases and fluids that would be useful to the study and prediction of large earthquakes and volcanic eruptions (e.g.^24–28^). ^3^He and ^4^He-based detectors are also useful for monitoring nuclear storage sites and facilities, both as an intrinsic detector and as a detection medium for neutrons (e.g. ref.^29^).

The trapping efficiency for ^3^He and ^4^He in these experiments can be estimated from their known concentration in dry air, sample volume, average net ^3^He and ^4^He PP (from Fig. 3 heat ramps), and volume of the instrument’s vacuum. These calculated efficiencies are 0.672 and 0.638%, respectively. The ^3^He/^4^He diffusion efficiency ratio in quartz glass is, on average, 6.72 E-03/6.38 E-03 = 1.05 ± 0.40. Both isotopes have essentially the same efficiency despite their disparate relative concentrations in air and temperature dependence. This ratio is similar to the diffusivity ratio reported for natural glasses by Trull^4^. It is important to note that the calculations assume that the integration times over the temperature history account for these differential diffusion effects in quartz glass, and similar integrations may explain why these differential diffusion effects were not previously recognized. Efficiency calculations are shown below.

Molar ^3^He concentration in air sample = n = PV/RT = (1 atm) R_a_ * [^4^He] * sample volume = (1 atm) 1.40 E-06 (5.22 ppm) (3.28 L) = 1.40 E-06 (5.22 E-06) (3.28 L) = 7.31 E-12 (3.28 L) = 2.40 E-11/[0.08206 (298)] = 9.81 E-13 moles. Molar ^3^He concentration in inst. vacuum = n = PV/RT = (0.97 ± 0.34 E-12 Torr [Average and standard error of net integrations for four highest power heat ramps in Fig. 3.]) (1 atm/7.60 E + 02 Torr) (40.5 C.F. [C.F. = conversion factor calculated between the NIST-traceable ART mass spectrometer-390-gauge PP and those recorded by the MKS Spectra mass spectrometer. Conversion factors were calculated from highly linear regression slopes of PPs for in-common H_2_ and ^4^He, and extrapolated for ^3^He at intermediate mass/charge.]) (3.12 L)/RT = 6.60 E-15 moles. Absolute ^3^He diffusion efficiency = 6.60 E-15 moles/9.81 E-13 moles = 6.72 E-03 or 0.672**%**.

For ^4^He, these calculations are: Molar ^4^He concentration in air sample = n = PV/RT = (1 atm) [^4^He] * sample volume = (1 atm) (5.22 ppm) (3.28 L)/[0.08206 (298)] = 5.22 E-06 (3.28 L)/24.45 = 7.00 E-07 moles. Molar ^4^He concentration in inst. vacuum = n = PV/RT = (5.91 ± 2.23 E-07 Torr [Average and standard error of net integrations for four highest power heat ramps in Fig. 3.]) (1 atm/7.60 E + 02 Torr)(45 C.F. [C.F. = conversion factor calculated between the NIST-traceable ART mass spectrometer-390-gauge PP and those recorded by the MKS Spectra mass spectrometer. Conversion factors were calculated from highly linear regression slopes of PPs for in-common H_2_ and ^4^He, and extrapolated for ^3^He at intermediate mass/charge.]) (3.12 L)/RT = (7.78 E-10 atm) (45 C.F.) (3.12 L)/[0.08206 (298)] = 1.09 E-07/24.45 = 4.46 E-09 moles. Absolute ^4^He diffusion efficiency = 4.46 E-09 moles/7.00 E-07 moles = 6.38 E-03 or 0.638%.

We conclude that the heat ramps performed with this prototype were not highly efficient at transferring He isotopes from dry air into the instrument’s vacuum. Nevertheless, these experiments demonstrate an effective measurement of the isotope ratio at air concentrations that is temperature dependent, and suggest that a previously unknown, relatively low-power concentration method may exist for transferring ^3^He from the atmosphere, heretofore thought too dilute as a potential resource of this rare isotope. The ^3^He enrichment window in quartz glass occurs within ca. 150° to 450 °C, with even narrower peaks within this range that are presently poorly resolved but strongly suggest quantized vibrational entrance temperature states are driving its diffusion via tunneling. Procedural changes such as greater integration time within the temperature window and higher applied sample pressure, plus design changes such as greater glass surface area and thinner wall thickness would improve the absolute transfer efficiency (c.f. ref.^1^). The transparency of the glass transfer medium and the low temperatures required suggest a passive, solar-heated surface array as a concentration facility, requiring little effort except for occasional sweeps of the vacuum chamber to tantalum getters.

## Methods

Experiments were made on a compact, elongate vacuum apparatus that featured two vacuums, a high to UHV vacuum chamber and a sample chamber surrounding a glass inlet that could be evacuated (Fig. 1). We used a cylindrical, “domed” quartz glass adapter assembly sited within a sample chamber where air was introduced via a gas inlet valve and maintained at constant pressure by pumping or thermally pressurized by valve isolation upon heating. Gases diffuse through the glass and pass into a static UHV Measurement System that contains both mass spectrometers, vacuum pumps, and total-pressure measurement device. The entire system is constructed with vacuum components consisting of commercial Conflat™-type stainless steel (SS) flanges and housed in commercial grade 304-stainless steel components with hydrogen partial pressure minimized by Non-Evaporable Getter (NEG) pumping.

We used a high-purity (99.995% SiO_2_) GE quartz glass cylinder of 2.5-inch (6.35 cm) diameter by 7 inches (17.78 cm) length that was fused with lower-temperature borosilicate glass and Kovar™ to a 4.5 inch (11.43 cm) Conflat™ SS vacuum flange. The glass vacuum fitting was 2 mm in wall thickness. The glass surface area available for the experiment was 532 cm^2^. Heat was applied with a Samox^®^-coated high-temperature heat tape with power controlled by a Variac™ variable autotransformer. Temperature was monitored using an Omega thermocouple (TC) probe placed to within a few mm of the glass surface via an Agilent data logger with data simultaneously fed into the MKS Microvision-2 quadrupole mass spectrometer (QMS) through a custom TC interface. The enclosed, vacuum-tight SS sample chamber had a 4-pin electrical feed-through and welded inlet and outlet SS tubing for both pumped and static (no flow) gas options. Lab air relative humidity ranged from 50 to 60%, and was dried by re-circulated flow through an indicating chemical desiccant trap prior to inlet into the sample chamber.

A SAES Getters NEG pump and a Gamma Vacuum noble diode ion pump maintained high to UHV conditions after bringing the system within range with a turbo-roughing pump station. The vacuum quality and other gases of interest, specifically H_2_ and ^4^He, were monitored with an MKS ART (Auto-Resonant Ion Trap) MS coupled to a NIST-traceable, full-range vacuum gauge (MKS model 390) running on the same PC computer clock as the QMS. The ART MS computes absolute partial pressures by a ratiometric technique, combining the relative intensity ratios of a chosen peak response to the total MS response with the total pressure measured simultaneously. The QMS produced factory-calibrated peak response, which we found was offset from the ART by a linearly-calculable factor used for relating the two MS response values at in-common masses 2 and 4. The MKS Microvision-2 QMS was modified for this application to work at very high frequency in the mass range 1–6 amu, and was thus optimized for sensitivity at relatively high mass resolution.

Scan samples of laboratory air reported herein were continuously collected as near to complete-cycle heat ramps from ambient laboratory temperatures to maximum temperatures determined by the voltage setting on the Variac. The heating up-ramps were logarithmic curves (R^2^ ≥ 0.95) that reached 200 °C in 2.8 minutes at V = 130. Reaching this temperature would take over 15.6 hours at V = 25. After heating for one hour, cooling curves were exponential after line power was cut off (Figs. 2 and 3). Operation of a high-vacuum valve that isolated the ion pump rapidly cleared He, Ne and Ar which had accumulated in the high vacuum chamber. Hydrogen was continuously pumped by the NEG, along with any reactive species such as H_2_O and CH_4_.

The modified MKS quadrupole MS could not resolve contributions to the mass-3 peak from well-known interfering ^3^He isobars of HD, ^3^H (tritium) and H_3_ (triatomic hydrogen). For these experiments, we assumed the contribution from environmental tritium was negligible and concentrated on resolving the HD plus H_3_ isobars. HD is 0.01% of environmental hydrogen on Earth but may suggest a different relative abundance in the vacuum components, as shown in the Supplementary Information section. We used two resolution methods. The first was statistical linear regression from plots of mass-2 versus mass-3, where the abscissa (x-axis) is the H_2_ peak response and the ordinate (y-axis) is the combined peak response from ^3^He and HD. A positive intercept at zero H_2_ will be residual ^3^He, as HD and H_2_ co-vary with an assumed constant ratio (e.g. ref.^30^). The second method, called herein Adjusted Ionization Mass Spectrometry (AIMS) (TIMS of ref.^31^), works by lowering the ionization potential from the standard 70 eV to less than 30 eV, effectively eliminating any ^3^He, as helium is not responsive beneath this ionization threshold. The lower ionization mass-3 response is therefore residual HD and H_3_ (plus any tritium), to be subtracted from the higher ionization peak with combined ^3^He, HD, H_3_, and ^3^H response. A co-variance with R^2^ = 0.79 (n = 44) was found for these two methods as “blind temperature ramps”, where QMS scan measurements were recorded immediately upon cool-down following 1-hour heat ramp completions. Comparison of lab air ^3^He/^4^He ratios determined by these two methods were within a factor of 2 of the accepted air ratio of 1.40 E-06 at ramp maximum temperatures above 300 °C (refs^27,28,32^; see Supplementary Information).

## Supplementary information

Supplementary Info.

## Data Availability

The datasets generated during and/or analyzed during the current study are available from the corresponding author on reasonable request.
