# Supplementary material for: Differential Diffusion of Helium Isotopes in Glass, Quantum-tunneling 3He Enrichment, and Portable 3He/4He Monitoring of Mantle Processes
Source: Sci Rep. 2019 Mar 26;9:5213. doi: 10.1038/s41598-019-41360-5 (PMC6435696; doi:10.1038/s41598-019-41360-5)
Supplement: Supplementary file 1 — Supplementary Info. [file 41598_2019_41360_MOESM1_ESM.docx]

**Supplementary Information**

**Differential Diffusion of Helium Isotopes in Glass, Quantum-tunneling ^3^He Enrichment, and Portable ^3^He/^4^He Monitoring of Mantle Processes**

Gary M. McMurtry^1^, James R. DeLuze^2^, David R. Hilton^3†^, and James E. Blessing^4^

1. Dept. of Oceanography

School of Ocean and Earth Science and Technology

University of Hawaii, Manoa

Honolulu, HI 96822

USA

2. Fusion Energy Solutions of Hawaii

611 University Avenue, Apt. 301

Honolulu, HI 96826

USA

3. Scripps Institution of Oceanography

University of California, San Diego

9500 Gilman Drive

La Jolla, CA 92093-0244

USA

4. MKS Instruments, Inc.

Mass Spectrometry Solutions

3635 Peterson Way

Santa Clara, CA 95054

USA

† Deceased January 7, 2018.

Figure S1 displays mass-2 versus mass-3 plots for two of the static-air heat ramps shown in Fig. 3. They display nearly closed hysteresis curves, and also reveal the narrow ^3^He enrichment window passed during the warm-up portion of the heat ramp. For V=90, exclusion of the ^3^He diffusion spike data in the warm-up trend yields the air ratio as intercept, whereas the cool-down trend yields a 9 to 14 times higher value. Other static air heat ramps show similar trends. For V=130, the warm-up trend yields an R/R_a_ ratio within the error for that of air. Upon cool-down, two trends are evident: (1) a steep, highly linear trend with negative intercept that likely results from HD in excess of the air HD/H_2_ ratio (green shading); and (2) a lower temperature trend that yields an R/R_a_ ratio about 10 times the air ratio.

The ^3^He enrichment temperature window passed upon warm up is intersected again as the glass cools down, but ^3^He enrichment is not apparent. An explanation for its apparent absence may be in the anomalous amount of HD (compared with its expected ratio to H_2_ in air) that diffuses through the glass at the beginning of the cool-down cycle (Fig. S1), which may mask any excess ^3^He and suppress the ^3^He/^4^He and R/R_a_ ratios in those portions of the heat ramps (Fig. 3). We note that the positive intercepts of the lower portion of the cool-down trend yield intercept R/R_a_ ratios that are higher than that of air (Fig. S1). These positive intercept values suggest that ^3^He is indeed enriched over the air ratio during the cooling of the heat ramp, and the enrichment persists as higher ^3^He/^4^He backgrounds than air after the sample heat ramps.

Figure S2 presents a generalized correlation of the two HD-^3^He determination methods used. For these analyses, the Adjusted Ionization Mass Spectrometry (AIMS) data were collected “blind”, by recording the QMS scans immediately after heat-ramp cool down to near ambient temperature. Brown points represent atmospheric pressure air samples. In cases where a few hours elapsed without pumping the high vacuum with the ion pump before re-recording these data, an enrichment of ^3^He in the vacuum background produced higher ^3^He/^4^He ratios for these samples (orange arrows). Lower air sample pressures (green points) generally produced higher ^3^He/^4^He ratios more representative of the NEG high vacuum. Blue points represent variously pumped NEG vacuum backgrounds (e.g., ion pump, turbo-rough pumping).

Multiple runs of standards and analyzed samples using the AIMS method (TIMS of ref. 31) yields a tight distribution of (HD+H_3_)/H_2_ of 2.36±0.085 E-04 (n=16), probably all generated from filament ionization inside the high vacuum. The natural abundance ratio of HD/H_2_ is 1.15 E-04 (<https://en.wikipedia.org/wiki/Isotopes_of_hydrogen>). The slightly over a factor of 2 abundance ratio measured likely results from H_3_ that is often resolved in approximate equal abundance to HD in high-resolution mass spectrometers, but not resolvable in the MKS mass spectrometer. Further experimental results from instrument blanks run as pure dry nitrogen in the sample chamber after previously purging the glass of all helium, yield values for (HD+H_3_)/H_2_ of 2.33±0.057 E-04 (n=2), in close agreement with the runs of standards and analyzed samples. A one-to-one plot of a selection of these analyzed samples and standard gases versus the recommended values from conventional mass spectrometry is displayed as figure S3. To obtain the best fit curve of y = 1.08x – 0.42 (r^2^ = 0.983, n= 18), we used an instrumental correction of y = y’/1.83 - 2.45. The cause of this multiplicative correction is presently unknown.

Figure S4 presents a more restricted temperature dataset for dry laboratory air where ramps to higher and lower temperature were not collected, and, by running the noble ion pump during the heat ramps, nor were any fractionated collections in the high vacuum allowed. After the glass was at the target temperature, we closed off the ion pump and allowed a brief period, usually under an hour, for the noble gases to return to more detectable partial pressures prior to sampling. These results continue to show the temperature dependence of the ^3^He/^4^He ratio, which is inverse with temperature for ^3^He, in agreement with the results of refs. 11-15 in modified graphene, and positive with temperature for ^4^He, the latter in agreement with the previous work on pure glass (1-3). In these experimental runs, the Rc/Ra** values approach that of air at temperatures above ca. 500°C (Fig. S4). A reasonable explanation of this trend is the lower-temperature enrichments in ^3^He result from this isotope’s storage within the glass matrix (e.g., ref. 10) with subsequent release upon removal of the noble ion pump exposure to the high vacuum.


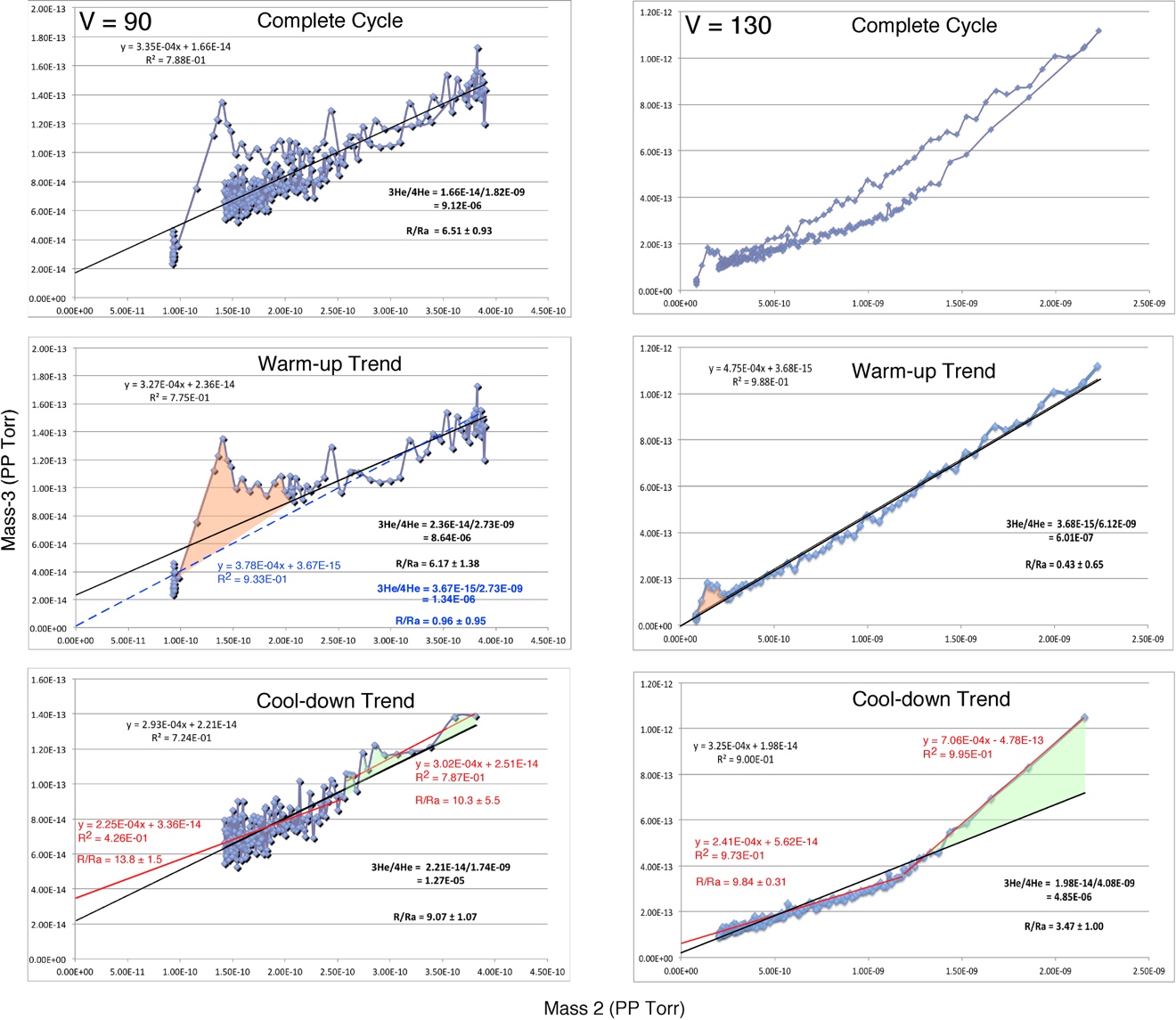


**Fig. S1.** Plots of mass-2 (H_2_ partial pressure, PP) versus 3 (combined HD-H_3_-^3^He mass-3 peak) for static air heat ramps to maximum 370°C (left; V = 90 in Fig. 3) and to maximum 470°C (right; V=130 in Fig. 3). Lower panels show warm-up and cool-down trends, with window for enhanced ^3^He diffusion indicated by orange shading. ^4^He values for the complete cycle, warm-up and cool-down were chosen as the average, peak, and final run PP, respectively.


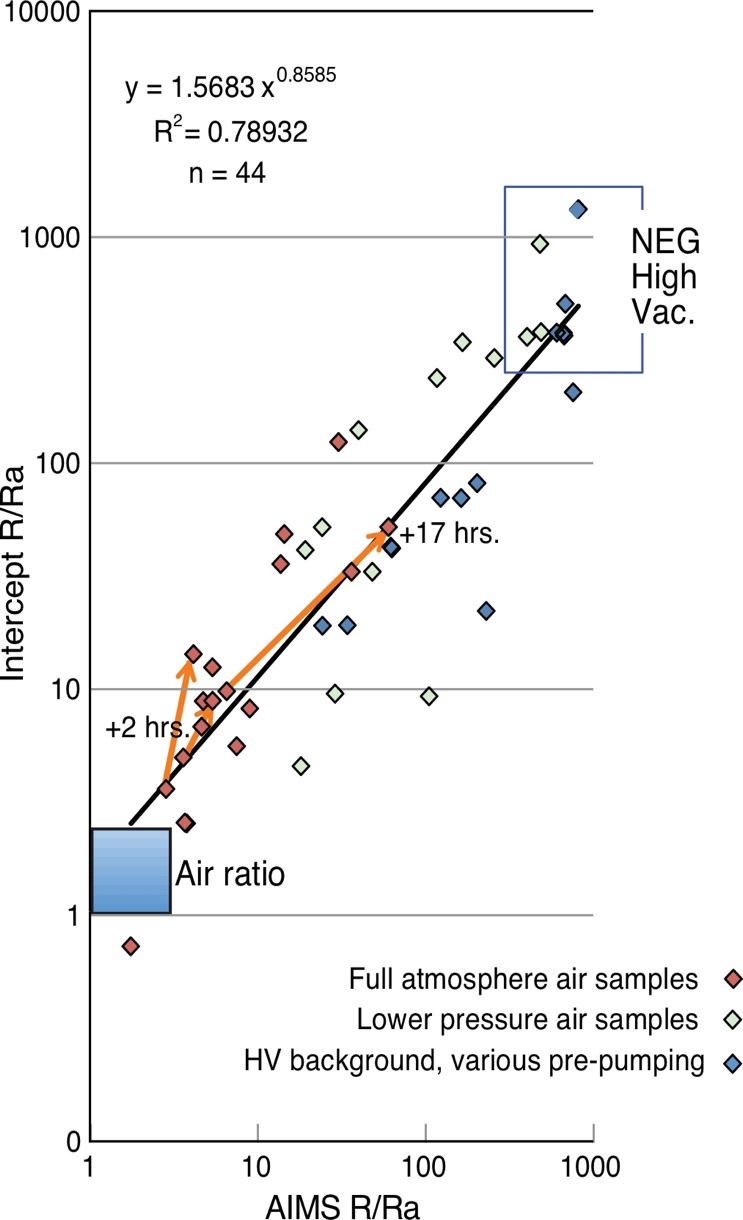


**Fig. S2.** Comparison of the two HD-^3^He mass resolution methods used. Brown points are atmospheric pressure lab air samples. Green points are various lower-pressure air samples, and blue points represent variously pumped NEG vacuum backgrounds. Arrows indicate new values obtained for those air samples after prolonged exposure to the NEG high vacuum.

**Fig. S3**. Plot of known or accepted value versus observed Rc/Ra from this study, using the field portable helium isotope monitor instrument. The AIMS method was used with three prototype instruments made and utilized to date. Albert is a bench-top unit described in the text; Edward and Lyle are intended as identical, relatively compact field-portable units, with smaller 1.5-inch (3.81 cm) diameter cylindrical quartz adapter flange and sample chamber (28). The Helium Standard of Japan (HESJ) is a known artificial gas mixture for isotopic calibration (32). Salton Sea mud pots CO_2_-rich gas was provided from D. Hilton lab, SIO. The Mammoth samples were nearly pure CO_2_-rich gas collected from the soil at Horseshoe Lake site (HLS), Mammoth Mountain, CA; conventional values from USGS Noble Gas Lab, Denver Federal Center, A. Hunt, analyst. The Kilauea Summit samples are air-corrected values from gas samples collected at Sulphur Banks solfatara, D. Bergfeld and A. Hunt, analysts. The air means are from various runs of the three prototypes, three of lab air and one from automatic collection at HLS. Further information can be found in McMurtry et al., 2019 (ms. in prep.).


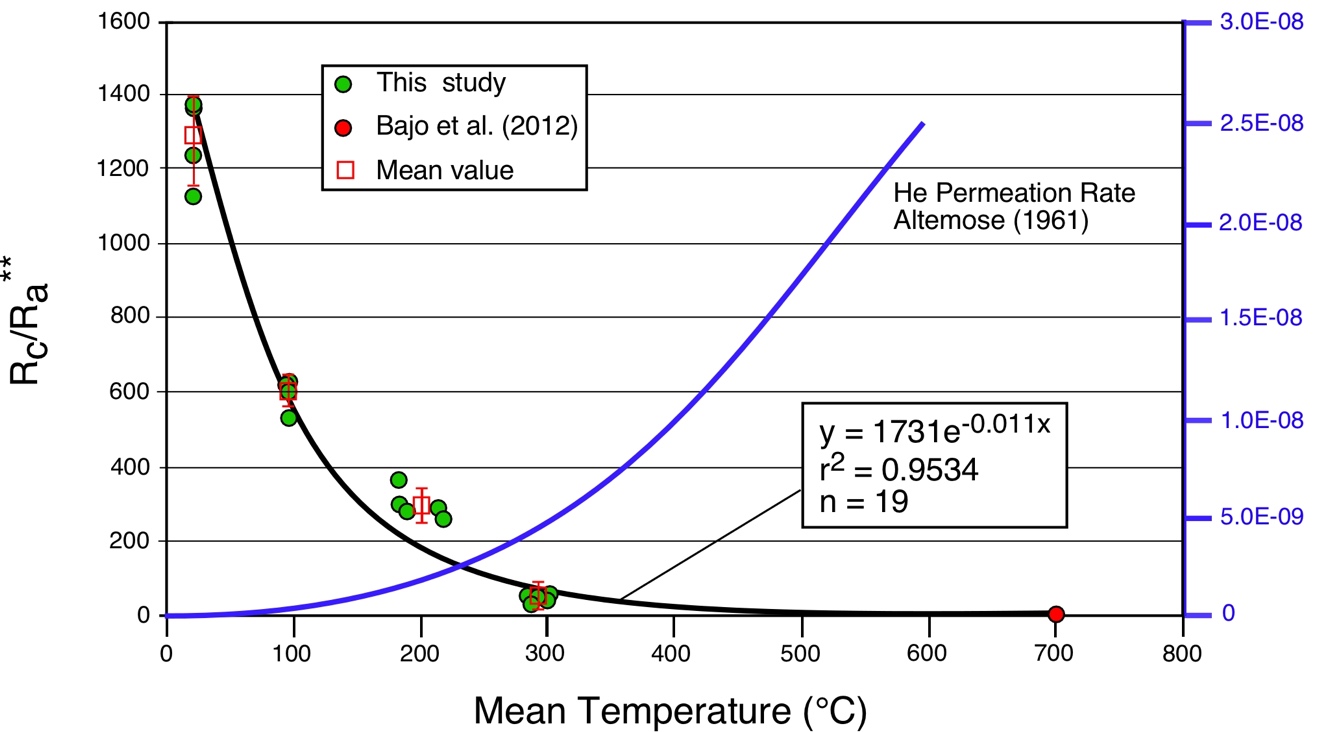


**Fig. S4.** Trend of AIMS air ^3^He/^4^He ratios versus mean run temperatures, where Rc/Ra** = corrected AIMS ^3^He/^4^He ratio normalized to air ratio. These data were collected as quasi-isothermal runs with noble ion pumping during the warm up ramp to eliminate any ^3^He/^4^He enrichment in the glass at lower temperatures prior to the run. Also shown is the trend of ^4^He permeation rate in pure silica glass versus temperature experimentally determined by Altemose (3).
